# Brain Connectivity Networks and the Aesthetic Experience of Music

**DOI:** 10.3390/brainsci8060107

**Published:** 2018-06-12

**Authors:** Mark Reybrouck, Peter Vuust, Elvira Brattico

**Affiliations:** 1 Faculty of Arts, University of Leuven, 3000 Leuven, Belgium; mark.reybrouck@kuleuven.be; 2 Department of Art History, Musicology and Theater Studies, IPEM Institute for Psychoacoustics and Electronic Music, 9000 Ghent, Belgium; 3 Center for Music in the Brain, Department of Clinical Medicine, Aarhus University & The Royal Academy of Music Aarhus/Aalborg, 8000 Aarhus, Denmark; petervuust@gmail.com

**Keywords:** neuroaesthetics, music processing, reward brain system, connectivity network, default mode network

## Abstract

Listening to music is above all a human experience, which becomes an aesthetic experience when an individual immerses himself/herself in the music, dedicating attention to perceptual-cognitive-affective interpretation and evaluation. The study of these processes where the individual perceives, understands, enjoys and evaluates a set of auditory stimuli has mainly been focused on the effect of music on specific brain structures, as measured with neurophysiology and neuroimaging techniques. The very recent application of network science algorithms to brain research allows an insight into the functional connectivity between brain regions. These studies in network neuroscience have identified distinct circuits that function during goal-directed tasks and resting states. We review recent neuroimaging findings which indicate that music listening is traceable in terms of network connectivity and activations of target regions in the brain, in particular between the auditory cortex, the reward brain system and brain regions active during mind wandering.

## 1. Introduction

Traditionally, music has been studied as a human artifact, focusing to a certain degree on the structural analysis of the score and on the historical birth and fortune of the compositions. Though legitimate and useful, the structural and historical approaches take only partially into account the listener’s experience while listening [1]. As a result, there has been a paradigm shift in some subfields of music research, which can be described as an “experiential” and “pragmatic turn” [2,3]. A first instigation was given by the pioneers of early cognitive musicology who claimed that music is above all a human experience rather than a petrified structure to be studied outside of the time of actual unfolding [4]. A huge body of research has followed since these early days, with a major focus on music and cognition and the computational modelling of musical knowledge [5,6,7,8,9,10]. This approach, however, was still more cognitive than experiential (for applications of experiential and phenomenological views to neuroscience, see [11]). With the recent development in neuroimaging, cognitive neuroscience and visual neuroaesthetics, a novel approach to music providing a phenomenological brain-based framework for the aesthetic *experience of music* could finally be introduced [12,13,14,15,16].

From the perspective of the mind, an aesthetic experience can be defined as a kind of exploratory behavior [17], integrating several levels of processing, such as the perceptual, action-related, cognitive, affective and evaluative ones. In neuroscience research, though, scholars have mainly focused on stimulus-based perceptive and cognitive aspects of the experience of listening to music. Yet, there is accumulating evidence that aesthetic, namely subjective, affective and evaluative processes are at play during listening and hence should be considered as well for systematic investigation [13,14,18,19,20,21,22,23]. This paper is inspired by this emerging field of neuroaesthetics [21,24,25,26,27,28,29] and aims at drawing a coherent summary of the most recent findings relating the *musical aesthetic experience* with the underlying brain patterns. The emerging picture is that (aesthetic) music listening is associated with neural connectivity patterns rather than a one-to-one mapping to single brain structures.

Hence, we start from the conception of music listening as a network-based brain function, and then we bring together insights from neural connectivity and neuroaesthetics. We argue that the combined contributions from neuroimaging, network science and connectomics should be able to provide the much-needed framework for studying the role of the human brain while dealing with music. The first results point into the direction of the involvement of the medial structures of the brain related to the default mode network during listening to favorite music. Another set of results highlights a convergence between aesthetic responses and the connectivity of the reward circuit with the inferotemporal cortex and of the default mode with audiomotor networks. A first overview of the studies is given in Table 1 with a short description of the method, participants, and major findings of these studies.

## 2. Neuroaesthetics of Music

Experimental studies of brain mechanisms involved in the appreciation of art and aesthetics have popped up abundantly in recent years. They are part of a small but active research program inside the field of neuroscience that focuses on the neuroscience of aesthetics. *Neuroaesthetics*, as the term has been coined by Zeki [42], is an inquiry into the neurobiological substrates of an aesthetic experience [27]. As a field, it is gathering force, not by providing a mere catalogue of brain regions that are related to aesthetic experience, but by encompassing the perception, production and response to art, as well as the interactions with these objects, scenes, or events that evoke intense emotions [24]. As an experimental science, it emphasizes the neural underpinnings of these experiences [28,43] in contrast to the processing of other, more neutral stimuli. It has recently been argued that a number of fundamental characteristics of the human nervous system are responsible for our sensitivity to global sensory properties [44,45]. This was also the main claim of Bell’s theory of *significant form* [46], which stated that certain combinations of lines and colors—at least for the visual domain—should arouse an aesthetic emotion, and in a universal way, independent of time, culture, and historic era. Zeki [29], who provides a modern version of this theory, found that the experience of beauty correlates with neural activity in the A1 field of the *medial orbito-frontal cortex* (mOFC)—a part of the emotional brain—regardless of stimulus source and of culture or education of the perceiver. Moreover, this neural activity seems to be detectable and quantifiable, which makes it apt for empirical investigation.

In neuroaesthetics, the perception of artworks is related to definite preferential activation patterns of domain-specific early sensory areas, which may lead to activations of attention- and motivation-related brain areas as well as an experience of emotion, beauty, and preference [21,22,38]. Perception of artworks might then entail positive feelings of liking and enjoying, which results in a combination of bottom-up processes involving sensory prediction and anticipation, fast emotional reactions, and reward representation, and top-down processes, such as affective self-monitoring, intentionality, cognitive mastering, and evaluation [18,47,48]. All these processes take place in distinct cortical brain areas (anterior cingulate, orbitofrontal, and ventromedial prefrontal) as well as subcortical striatal regions belonging to the reward circuit, namely the caudate nucleus, substantia nigra, and nucleus accumbens, as well as a number of molecular regulators of this circuit (in particular, dopamine and opioid) [24,27]. Neuroaesthetics, in this view, is not concerned with particular artworks or features but rather with the way they are experienced when people take an *aesthetic attitude* towards them, namely an intention targeted at focusing attentively on an object for finally feeling an emotion and/or issuing a judgment [45,49,50]. The studies focusing on bottom-up artwork-related processes, however, do not conflict with those looking into top-down experiential factors, since both bottom-up and top-down processes converge in explaining how artistic techniques and resources have been devised to catch our attention, interest and appeal, so as to engage some neural processes which invoke rewarding sensations [19,22,51]. 

The sources of an aesthetic experience can be numerous—examples are nature scenery, food, music, faces, smells and many others—but the evolution of the neural mechanisms that endowed humans with the capacity to engage in rewarding sensations in different modalities (visual, auditory, olfactory, gustatory, tactile, and kinaesthetic) is still elusive to some extent [52]. Humans, moreover, appear to be unique as biological organisms in their aesthetic orientation towards rewarding stimuli, but the involved brain regions seem to be involved also in other kinds of experience. This could suggest that aesthetic experiences rely on neural mechanisms that are nonspecific and general, such as attention- and motivation-related processes, which are shared also with some of their close primate relatives [28,52]. On the other hand, aesthetic experiences rely on the interaction between domain-general neural processes and artwork-derived sensory processes. To reveal the nature of this interaction, we need to implement an interdisciplinary framework including psychological, neuroscientific, and biological approaches [45]. 

Inspired by the broader field of neuroaesthetics (focusing most on visual figurative art), recent proposals have put forward a *neurobiological* and *psychobiological approach* to music viewed as an art form [12,14,53,54]. This approach is intrinsically characterized by emotional, aesthetic, and evaluative processes, rather than focusing on cognitive representation and processing of musical structure. A musical-aesthetic experience, in this view, is described as an experience in which an individual immerses herself/himself in the music, dedicating attention to perceptual, cognitive, and affective interpretation based on the formal properties of the perceptual experience [14]. As such, and similarly to other art forms [22,25,55], the subjective experience of music is linked to three distinct cognitive processes: an emotional experience, an aesthetic judgment of beauty or other formal qualities attributed to the artwork, and a verdict of liking or preference [14,56]. In a review article by Brattico et al. [13], findings obtained using brain research methods have been summarized and ordered to delineate a putative chronometric succession of neural/psychological processes leading to the aesthetic experience of music (see also [16]). The picture obtained was complex and yet undefined since studies were sparse. Some progress, however, has been made in the past five years, especially in relation to visual neuroaesthetics, where the results converge into a view of aesthetic experience as a composite of several processes that rely on a number of brain structures working in synchrony (see e.g., [57,58]). In general, cognitive neuroscience has moved towards understanding brain architecture and function as a complex (small-world) network linking brain structure to functional brain specificity and integration [59]. Hence, the original approaches focused on one-to-one brain mapping may have to be replaced by network views of brain structures working in concert for executing complex tasks.

## 3. Network Neuroscience and Connectomics

In the past decade, the neural correlates of brain functions have been searched from the interactive communication between brain structures rather than from independent activity in each structure. This search for “connectivity” exploits algorithms and concepts developed within the mathematical field of network science. The novel research field of “connectomics” has the final goal to generate a complete map of all neural connections on a template brain with its functions, by describing the brain as a large structural network made up of neural connections, consisting mainly of white matter tracts and neural units consisting of grey matter. Network neuroscience, as a new field of research, relies on new techniques and analysis methods, such as diffusion tensor MRI, tractography, stochastic dynamic causal modeling (DCM), and whole brain computational modelling [60] for in vivo examination of anatomical and functional interactions on a whole-brain scale [61]. The goal is to obtain information on the amount and direction that activity patterns in a particular brain region exert over another [62]. The technique of tractography has been very important in this regard. It can be used to examine the *anatomical connectivity* between different brain areas, namely how white matter fibers connect each brain region [63]. FMRI and MEG, in turn, can be used to study *functional connectivity*, which can be defined as the temporal dependence of neuronal activity patterns of anatomically separated regions. It can be measured by the level of simultaneous coactivation of fMRI or MEG time-series in different locations of the brain [64].

Connectomics research puts forward two alternating brain network systems, which have been labelled as “task positive” and “task negative” or “resting networks” [65]. The former is recruited when there is active engagement in goal-directed tasks with attention to the world and evaluation of the salience of external stimuli. It has also been coined as the “looking out” or “executive function” system. The latter is related to the spontaneous low-frequency BOLD fluctuations of the resting brain [66,67,68]. Whereas the spontaneous fluctuations during resting state connect distant parts of the brain and hence evidence the presence of the brain’s consistent network organization similar to what is found during the performance of sensorimotor and cognitive tasks, a specific set of regions is what specifically characterizes rest or the brain’s default mode operation, DMN. Hence, it is supposed that DMN is a cortical system that is responsible for self-estimation and cognitive appraisal. The core regions of the DMN are all along the midline of the brain in the parietal and frontal lobes (prefrontal midline regions and the posterior cingulate cortex (PCC) and retrosplenial cortex) with an architecture that reveals interconnected subsystems with key hubs in the PCC that are connected with the medial temporal lobe memory system [66]. Key (hub) regions of the frontal DMN overlap with the hedonic network (anterior cingulate and orbitofrontal cortices), namely connected brain regions responsible of pleasurable sensations and feelings [69]. Moreover, these hedonic network regions have a relatively high density of opiate receptors, which explains to some extent the connectivity of these networks.

Functional connectivity, moreover, is not fixed but is to be considered as a dynamic community structure that is modifiable by recent experiences and learning histories, both within and between the networks they recruit [62]. Such community organization can change smoothly over time and is modulated both by exogenous stimulations as well as spontaneous activity during rest [70]. It has been hypothesized that the obtained connectivity patterns are the result of music-related mentation, outside of the context of actual performance [71]. Recent methodologies allow the tracking of how connectivity states dynamically change over time during the course of music listening [72]. These methodological advances open up new avenues for understanding the temporal succession of the underlying neural network processes.

The study of brain connectivity during music listening is an emergent field. Three major strands of research seem to crystallize up to now: (i) task-related connections, that is, those connections that are ongoing while listening to or making music, (ii) resting state connections, namely those that are active before or after musical activity while the brain is at rest, and (iii) the relations between task-related and resting states. For our present study, we focus mainly on the first and third of these three strands in relation to music aesthetics.

## 4. The Default Mode Network during Music Listening

The DMN is one of the brain circuits that are most studied in neuroimaging. It is believed to be a neural circuit that constantly monitors the sensory environment since it displays high activity during lack of focused attention on external events and decreases its activity during attention-demanding tasks [73]. It seems to function as a toggle switch between outwardly focused mind states and the internal or subjective sense of self [41,65]. Hence, the DMN can be understood in two ways: as a kind of intrinsic “activation” which is related to specific ways of cognitive functioning, or as a “deactivation” during other goal-directed tasks. As to the first, activations in the DMN have been reported during goal-directed tasks which involve introspective, self-referential thoughts and socio-emotional perspectives, such as empathy and levels of self-awareness [65,66,67,74]. As to the deactivation, it has been shown that the DMN is down-regulated during external attention-demanding tasks, and the efficiency of this down-regulation has been found to predict also performance of these tasks in real time [65]. 

**Figure 1:**
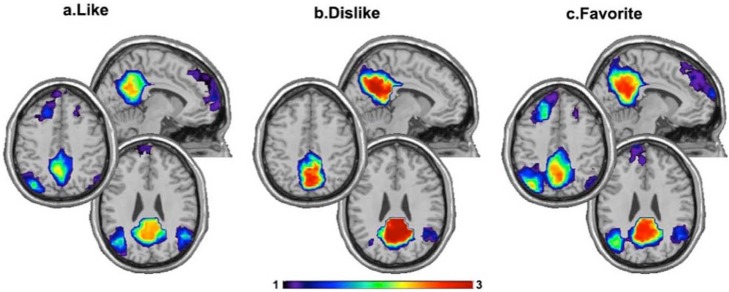
The figure illustrates the findings relating default-mode network (DMN) with aesthetic listening to music obtained by Wilkins et al. [41]: the structure of precuneus community within the default mode network depends on music preference. In the Liked and Favorite condition, the precuneus was consistently interconnected with lateral parietal and medial prefrontal cortex (**a**,**c**). When the music was disliked, the precuneus was relatively isolated from the rest of the default mode network (**b**). Color indicates the consistency of community structure for each voxel across subjects (Figure reproduced without any changes).

In the domain of visual neuroaesthetics, it has been convincingly shown that the DMN is involved during aesthetic contemplation [57,58]. Listening to music, for its part, has also the potential to alter the DMN connectivity of the brain, and hence it has been phenomenologically linked to the experience of mind-wandering and unfocused thought [33]. Preference for a musical genre, moreover, seems to dictate the connectivity that can be expected between brain regions. To our knowledge, the first study using network science with whole-brain fMRI data and looking at the relation between default mode network and aesthetic music listening was conducted by Wilkins et al. [41] (see Figure 1). More in detail, they applied network science methods to evaluate differences in functional brain connectivity when individuals listened to complete songs. They found that DMN was most connected when listening to preferred music and that listening to favorite songs can alter the connectivity between auditory brain areas and the hippocampus, a region responsible for memory and social–emotion consolidation. The DMN thus seems to be most connected when listening to preferred music, which points in the direction of DMN coupling with the hedonic networks as well. Connectivity findings related to DMN during music listening were also obtained by Taruffi et al. [40]. They noticed that listening to sad music was associated with higher centrality of “default mode” network hubs (e.g., medial orbitofrontal, anterior cingulate, and posterior cingulate cortex), namely with lower connections with other brain regions, as contrasted with listening to happy music. Also, sad music induced more mind-wandering as witnessed by a separate behavioral study performed on a separate large sample of young adult participants. Similarly, in Alluri et al. [30], free listening to whole pieces of music was showing high centrality values, specifically in non-expert participants as compared with professional musicians, who instead showed stronger connectivity in networks of brain regions dedicated to auditory processing and motor control.

Overall, the findings suggest a role of DMN during listening to music, especially with emotional and favorite music and particularly with naïve listeners. Citing Vessel et al. [58], we might speculate that the involvement of this brain circuit is linked with the importance of music for introspective thought and generally for the formation of self, identity, and cultural belongingness, especially in adolescence [75,76].

## 5. Brain Connections during the Experience of Musical Reward

Listening to music aimed at enjoyment or at a liking decision (such as when deciding to purchase a song) have been associated with activity and connectivity in the orbitofrontal cortex, the insula, the temporal pole, the nucleus accumbens, and the anterior cingulate cortex (see Table 1). Listening to self-selected emotional music, e.g., has been shown to provoke tight communication between a wide range of brain regions, including auditory, motor, and limbic systems [32]. In cases of peak pleasure during music listening, functional connectivity between the superior temporal gyrus (where the auditory cortex is located), the inferofrontal cortex (where hierarchical predictions for sounds are computed), and reward regions has been obtained. An important hub of the reward circuit is the nucleus accumbens which includes synapses functioning by dopamine release. In the seminal study by Salimpoor et al. [39], they asked participants to purchase using a variable sum of money or not to purchase 30–60 s clips of unfamiliar commercial music. The activity and connectivity in the dorsal (caudate) and ventral (right nucleus accumbens) striatum accounted for both the reported pleasurableness of the music and the amount of money people were willing to spend on it (nucleus accumbens being the main source of variation). Moreover, areas such as ventromedial and orbitofrontal prefrontal cortices, anterior cingulate cortex, amygdala, and hippocampus showed increased connectivity with the nucleus accumbens when unfamiliar music was experienced as pleasurable and desirable. Increased hemodynamic activity in superior temporal gyrus (and thus, the main auditory cortices) did not predict the reward value of music, whereas connectivity between subcortical and cortical regions, such as amygdala, superior temporal gyrus and prefrontal cortex, and the nucleus accumbens was found when music became more desirable and subjects were eager to spend more money on it. Hence, these results are paramount to evidence the need of studying functional connectivity along with or even rather than regional activations of single brain areas to stimuli.

Overall, there is a consistency in the findings linking aesthetic responses and the *reward system* with anatomical and functional connectivity between the auditory cortex and the mesolimbic reward circuit predicting musical enjoyment [32,37,38,39]. Furthermore, the findings on increased connectivity between reward-circuit structures (especially the nucleus accumbens) and the auditory-cognitive regions during enjoyment of music have advanced the hypothesis that higher order pleasures have originated from prosocial benefits [38,77]. Things, however, seem to be more complicated since these pleasure-invoking circuits are activated in some individuals but not in others. Therefore, understanding the neural bases of these differences between responding and not-responding individuals can be helpful to define the neural pathways by which sensory stimuli become rewarding. A possible answer is to be sought in the structural connectivity between auditory- and reward-processing regions in the brain which may give rise to specific aesthetic responses to music. This hypothesis is supported by findings of lower anatomical connectivity (measured with DTI) in those individuals experiencing less intense emotional responses to music when assessed with the Aesthetic Experience Scale in Music (AES-M), as compared with controls [38]. Converging evidence comes from the fMRI study by Martinez-Molina et al. [37] with music-anhedonic individuals, assessed by using the Barcelona Musical Reward Questionnaire (BMRQ). In that study, which used psychophysiological interaction method for studying connectivity from the seed region of the nucleus accumbens to the rest of the brain, anhedonic individuals had diminished striatal connectivity with the right auditory cortex as compared to hedonic individuals only for the task involving pleasure judgements on music clips. In turn, in a task where they had to bet on one of two numbers for receiving monetary rewards or not, their striatal connectivity did not significantly differ from that of controls.

The evidence available thus far suggests a considerable overlap between the brain connectivity mechanisms that are involved in the experience of fundamental pleasures, such as sex and food, and those involved in higher-order pleasures, such as monetary, artistic, musical, altruistic, and transcendent pleasures. This seems to imply that the hedonic brain systems for aesthetic experiences, which are important to happiness and positive mood, draw upon the same neurobiological roots that evolved for mere sensory pleasure [69]. They involve different, connected regions of the cerebral cortex—embracing the orbitofrontal cortex, insula, medial prefrontal, and cingulate cortex—as well as a number of subcortical regions, such as the ventral striatum and the amygdala. The latter, especially, seems to be quite important, as evidenced by brain lesion studies which report on the effect of impairment of the amygdala on the emotional response to music. Positive evidence exists for the involvement of the amygdala in the auditory recognition of emotions in vocal sounds—especially scream and yell—and speech prosody, and this structure appears to serve a multimodal role in the processing of emotions that are related to threat [78,79]. 

A mere hedonic approach, further, can be distinguished from eudaimonic happiness, in the sense that the latter implies at least a sense of engagement and meaning [80]. The distinction is related to the difference between liking (hedonic) and wanting (eudaimonic), with liking being mediated by opioid and GABAergic neurotransmitters in the nucleus accumbens shell and ventral pallidum, and wanting by dopaminergic activity in the nucleus accumbens core. Cortical structures, such as the cingulate and orbitofrontal cortex, may contribute to additional conscious modulations of the rewarding signal [69]. The mere “sensory pleasure”, however, can be viewed as an early affective reaction, somewhat similar to the concept of core affect [18]. To become a conscious emotion of enjoyment and to engender a liking judgment, a percept must be accompanied by an attribution of value mediated by personal association, knowledge, social constructs, and other top-down processes which are controlled by the prefrontal cortex and associate temporoparietal structures of the brain. “Wanting”, on the contrary, involves a motivational element by adding an incentive salience to raise attraction [81]. 

Care should be taken, however, not to generalize too much here as the reliability of these findings from data-driven approaches and functional connectivity analyses has been criticized to some extent. Typically, an fMRI study adopts a single method of analysis and statistical thresholding which can yield different results with the same stimulation paradigm than when another method is utilized [35,82]. Though brain structures related to visual, reward and auditory processing seem to show a consistent pattern of intrinsic spatial patterns of coherent neuroactivity during affective processing, the psychological processes (such as attitudes or evaluative judgments) associated with each experimental condition should be taken into account when it comes to the interpretation of the findings.

**Figure 2:**
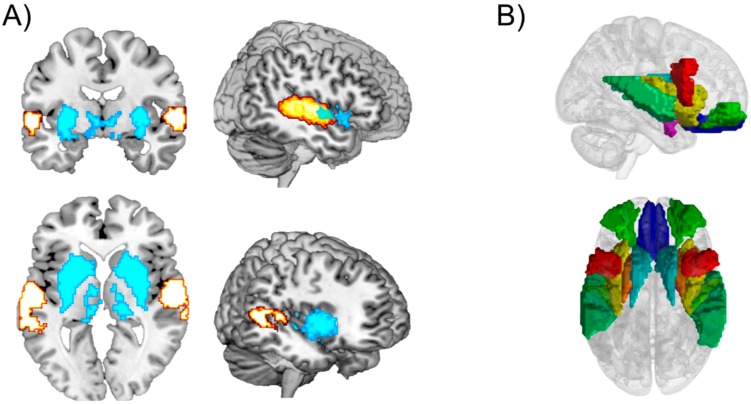
The figure presents the consistent results of functional connectivity between brain reward regions and auditory supratemporal regions during enjoyable music listening. (**A**) Brain maps plotting with MRICron on MNI template the two clusters showing the most consistent spatial patterns of coherent fMRI activity obtained during listening to emotional liked or disliked music by means of the consensus clustering paradigm, called binarization of consensus partition matrices (Bi-CoPaM) [35]. The cluster depicted in turquoise represents the anatomically and functionally connected regions of the reward system, including the striatum, globus pallidum, thalamus, insula, amygdala, and olfactory cortex, whereas the cluster depicted in yellow shows the connected regions of the auditory cortex including middle and superior temporal gyri, Rolandic operculum, and Heschl’s gyrus. (**B**) 3D maps plotted with fMRIToolbox (implemented at the University of Jyväskylä) showing with AAL parcellation the most consistent brain regions from the reward circuit, auditory system, and orbitofrontal cortex that are recurrently found connected during listening to pleasurable, liked music.

A novel data-driven analysis method—the consensus clustering paradigm, called binarization of consensus partition matrices (Bi-CoPaM) [83]—aimed at integrating results from several clustering methods such as clustering, independent component analysis (ICA), seed-based functional connectivity analysis, and inter-subject correlation analysis. This method has yielded some major reported findings: first, a cluster that includes the functionally connected cortical and subcortical structures of the reward system that responds selectively to liked, enjoyed music; second, a considerable impact of evaluative judgments on brain-networks related to music enjoyment [35]. Third, a down-regulation of auditory-limbic connectivity during conscious judgments of liking the music was found along with an increased connection between the audio-motor and attention-regulated regions. In case of non-evaluative but attentive listening, on the contrary, the results from this study pointed to auditory-limbic connectivity between the thalamus, superior temporal gyrus, amygdala, and parahippocampal gyrus, or between orbitofrontal regions or between supratemporal regions, insula and putamen. In case of evaluative judgments, a cluster of interconnections was found between regions related to cognitive processing of sound (middle temporal gyrus, rolandic operculum, inferior frontal gyrus) and regions related to observation of action and motor preparation (supplementary motor areas, precentral gyrus). Another cluster comprised higher-order structures involved in visual processing (cuneus, lingual gyrus, middle, inferior and superior occipital gyri and fusiform gyrus). It can thus be proposed that the subjective psychological state that can be captured with first-person measures is an important predictor of the emotion-related brain processes during listening to music [36]. Future studies could also answer the unsolved question of whether and how repeated aesthetic listening to music can shape neural connectivity (Figure 2).

## 6. Conclusions

The study of brain connectivity is on the recent agenda within the neurosciences of music. This approach has the potential to inform our knowledge about how the brain responds to music and decides if a musical piece is considered beautiful or not. As such, it can provide some of the much-needed neurological underpinnings of the neuroaesthetics of music and of the aesthetic experience of music. The latter, in particular, revolves around the neurobiological approach to music, integrating perceptual, cognitive, and affective levels of processing, soliciting the reward circuit, the DMN, and engaging the connectivity of the brain as a whole. Functional connectivity between brain networks related to aesthetic judgment, evaluative or even moral decision making, and the reward brain system has been shown already [32,38,39,69,77]. The link with the functioning of the DMN, however, is still subject to debate, both during task-related processes of actual listening and during resting states.

In general, efficient connections and organization between brain regions, which are well-measurable even during rest, have proven to be important for cognitive functioning and intellectual performance (according to the neural efficiency hypothesis): functional connectivity patterns may be used as a powerful predictor for cognitive performance [84] on one hand, and of disease on the other (e.g., [66]). Hence, the functional connectivity observed during music listening [30] could be linked to the beneficial impact of musical aesthetic experiences, such as listening to favorite songs, on well-being and even clinical conditions (see e.g., [85]). Even if these claims are still speculative to some extent, some recent empirical findings support this idea. For instance, a recent study using neuroimaging reported increased amplitude of low frequency fluctuations in the angular gyrus, a core center of the DMN, as well as higher connectivity with the pain modulatory network in patients with a chronic pain disease (fibromyalgia) only after listening to their favorite music and not to a control auditory sequence [31]. Even in 26 patients with schizophrenia, it has very recently been found that listening to music by Mozart in the course of 1 month of intervention has the effect of increasing resting-state functional connectivity from insula subregions to particularly cingular regions (regions belonging to the “salience” brain network). Moreover, these were significant predictors for the symptom remission after the music intervention [86].

Accumulating evidence thus indicates that music contributes to human well-being and health, in particular when music listening leads to an aesthetic experience. This highlights the need for more studies under the neuroaesthetic research agenda [12]. Contrary to the dominant approach of cognitive neuroscience of music, which has focused on playing music and the related neuroplastic changes, the effects of aesthetic listening on brain connectivity are still somewhat elusive up to now [14,19,30,49,52]. Listening, however, often thought as complementary to the act of performing, can in some instances replace the mere act of performing by providing a vicarious experience that relies on action-as-simulated rather than action-as-performed. It is obvious that listeners with an active training in music playing have access to this kind of simulation much more easily than non-musicians without musical motor repertoire. This sensorimotor coupling, however, is not the whole story. Initial findings reviewed here indicate that aesthetic listening, where the individual perceives, understands, enjoys and evaluates a musical event, is traceable in terms of network connectivity and activations of target regions in the brain. Whether and how this connectivity causally determines and predicts positive aesthetic responses, however, still remains to be understood.

## Figures and Tables

**Table 1:** Overview of previous research on music and brain connectivity. In the Method column, the order is as follows: stimulation design; measures; analysis method.

Study	Method	Participants	Major Findings
Alluri et al., 2017 [30]	Task-free music listening; fMRI; whole-brain graph-theory analyses	Musicians (*n* = 18) and non-musicians (*n* = 18)	Musicians primary hubs: cerebral and cerebellar sensorimotor regions, non-musicians: DMN-related regions.
Garza-Villareal et al., 2015 [31]	Resting state fMRI; BOLD signal	Non-musicians fibromyalgia patients (*n* = 22)	Increased amplitude of BOLD signal after listening; higher connectivity with right dorsolateral prefrontal cortex and left caudate; decreased connectivity with right anterior cingulate cortex, right supplementary motor area, precuneus and right precentral gyrus.
Karmonik et al., 2016 [32]	Task-free music listening; fMRI; graph network analysis	Non-musicians (*n* = 12)	Variation in functional connectivity after listening; most intense connections between brain areas were found for processing self-selected emotional music or culturally unfamiliar music.
Koelsch & Skouras, 2014 [33]	Mixed-block design; fMRI; eigenvector centrality mapping; functional connectivity analysis	Non-musicians, *n* = 20	Superficial amygdala, laterobasal amygdala, striatum, and hypothalamus function as computational hubs during joy evoked by music.
Koelsch, Skouras & Lohmann, 2018 [34]	Mixed-block design; fMRI	Non-musicians, *n* = 24	Anterior and posterior regions of auditory association cortex show functional connectivity with limbic/paralimbic, somatosensory, visual, motor-related, and attentional structures; primary auditory fields show strong functional connectivity with intra-auditory regions.
Liu et al., 2017a [35]	Mixed-block design; fMRI; consensus clustering with Bi-CoPam algorithm	Musicians (*n* = 13) and non-musicians (*n* = 16)	Brain structures related to visual, reward, and auditory processing show robust spatial patterns of coherent neuroactivity during affective processing.
Liu et al., 2017b [36]	Mixed-block design; fMRI; consensus clustering with Bi-CoPam algorithm	Non-musicians (*n* = 25)	Impact of explicit evaluative judgment on neural auditory-limbic connectivity during affective processing of music.
Martinez-Molina et al., 2016 [37]	Barcelona Musical Reward Questionnaire (BMRQ); skin conductance response (SCR); fMRI; psychophysiological interaction (PPI)	Non-musicians (*n* = 45)	Music anhedonic participants show selective reduction of activity for music in nucleus accumbens and decreased functional connectivity between right auditory cortex and ventral striatum.
Sachs et al., 2016 [38]	Survey data; behavioral, psychophysiological Measures; diffusion tensor imaging (DTI)	Experiencing chills to music (*n* = 10), not experiencing chills to music (*n* = 10)	White matter connectivity between sensory processing areas in superior temporal gyrus and emotional and social processing areas in insula and medial prefrontal cortex explains individual differences in reward sensitivity to music.
Salimpoor et al., 2013 [39]	Event-related design with decision-making after listening to music excerpts; fMRI; partial least-squares analysis	Not specified (*n* = 19)	During listening to purchased (vs. non-purchased) music clips, the nucleus accumbens increased its connectivity with superior temporal gyrus, orbitofrontal cortex, amygdala, ventromedial prefrontal cortex, anterior cingulate, and inferior frontal gyrus.
Taruffi et al., 2017 [40]	Mixed-block design; fMRI; ECM analysis	Not specified. Three experiments: *n* = 224; *n* = 140, *n* = 24	Sad music, compared with happy music, is associated with stronger mind-wandering and greater centrality of the nodes of the Default Mode Network.
Wilkins et al., 2014 [41]	Free listening; fMRI	Not specified (*n* = 21)	Circuit important for internally-focused thoughts, known as the default mode network is most connected when listening to preferred music.
